# Testing the effectiveness of a self-efficacy based exercise intervention for adults with venous leg ulcers: protocol of a randomised controlled trial

**DOI:** 10.1186/1471-5945-14-16

**Published:** 2014-10-03

**Authors:** Jane A O’Brien, Kathleen J Finlayson, Graham Kerr, Helen E Edwards

**Affiliations:** 1 School of Nursing, Institute of Health and Biomedical Innovation, Queensland University of Technology, Brisbane, Australia; 2 Institute of Health and Biomedical Innovation, Queensland University of Technology, Brisbane, Australia; 3 School of Exercise and Nutrition, Institute of Health and Biomedical Innovation, Queensland University of Technology, Brisbane, Australia; 4 School of Nursing, Institute of Health and Biomedical Innovation, Queensland University of Technology, Brisbane, Australia

**Keywords:** Venous leg ulcers, Exercise, Randomised controlled trial, Self-efficacy, Protocol

## Abstract

**Background:**

Exercise and adequate self-management capacity may be important strategies in the management of venous leg ulcers. However, it remains unclear if exercise improves the healing rates of venous leg ulcers and if a self-management exercise program based on self-efficacy theory is well adhered to.

**Method/design:**

This is a randomised controlled in adults with venous leg ulcers to determine the effectiveness of a self-efficacy based exercise intervention. Participants with venous leg ulcers are recruited from 3 clinical sites in Australia. After collection of baseline data, participants are randomised to either an intervention group or control group. The control group receive usual care, as recommended by evidence based guidelines. The intervention group receive an individualised program of calf muscle exercises and walking. The twelve week exercise program integrates multiple elements, including up to six telephone delivered behavioural coaching and goal setting sessions, supported by written materials, a pedometer and two follow-up booster calls if required. Participants are encouraged to seek social support among their friends, self-monitor their weekly steps and lower limb exercises. The control group are supported by a generic information sheet that the intervention group also receive encouraging lower limb exercises, a pedometer for self-management and phone calls at the same time points as the intervention group. The primary outcome is the healing rates of venous leg ulcers which are assessed at fortnightly clinic appointments. Secondary outcomes, assessed at baseline and 12 weeks: functional ability (range of ankle motion and Tinetti gait and balance score), quality of life and self-management scores.

**Discussion:**

This study seeks to address a significant gap in current wound management practice by providing evidence for the effectiveness of a home-based exercise program for adults with venous leg ulcers. Theory-driven, evidence-based strategies that can improve an individual’s exercise self-efficacy and self-management capacity could have a significant impact in improving the management of people with venous leg ulcers. Information gained from this study will provide much needed information on management of this chronic disease to promote health and independence in this population.

**Trial registration:**

Australian New Zealand Clinical Trials Registry ACTRN12612000475842.

## Background

Venous leg ulceration represents the most prevalent form of difficult to heal wounds affecting 1–3% of the population aged over 60 years [1]. This chronic condition not only affects the lives of those who suffer from them but requires a significant amount of health care resources [2]. Venous leg ulceration is defined as “an area of discontinuity of the epidermis and the dermis on the lower leg persisting for four weeks or more” [3]. Patients with venous leg ulcers report pain, sleep disturbances, wound odour, impaired mobility, altered body image, decreased vitality and disappointment with treatment leading to social isolation and an inability to work [4,5]. Furthermore, nearly half of the patients report insufficient levels of physical activity [6]. Although patients’ problems appear to be lifestyle related, advice to improve physical activity levels through adequate walking behaviour and leg exercises is not part of common wound management practice [7].

Failure of the calf muscle pump, from inadequate activity can result in sustained venous hypertension [8]. This theory is currently thought to be one of the main causes of venous leg ulceration [9]. The calf muscle pump is the primary mechanism to return blood from the lower limbs to the heart. The pathogenic steps leading from venous hypertension to ulceration are largely unknown. However, it has been found that the deficiency of the calf muscle pump function is significantly correlated with the severity of venous leg ulceration [10-12]. Our previous work in this area found that, after a 12 week home-based lower limb resistance exercise program, those exposed to the exercise intervention had significantly improved calf muscle pump function and range of ankle motion [11]. This is significant as an inefficient calf muscle pump and poor range of ankle motion are associated with prolonged healing [13,14]. This current study proposes to further explore the relationship between exercise and wound healing of venous leg ulcers. In comparison to previous work by the authors [11] there will be a larger sample size in this study and a focus on self-management for participants whilst addressing behaviour change theory in the development and implementation of the intervention. In addition this study aims to understand the role of exercise self-efficacy and outcome expectations in relation to adherence to exercise for a self-management home based exercise program for adults with venous leg ulcers.

Social Cognitive Theory (SCT) not only helps to explain and predict behaviour change, but also provides practical guidelines on how to design interventions [15]. Social cognitive theory has been widely used and tested successfully with health promotion interventions, including interventions for management of chronic disease such as multiple sclerosis and fibromyalgia [16,17] and health promoting behaviour in older adults [18,19].

Central to SCT is the concept of self-efficacy (SE) which is defined as *“people’s judgements of their capabilities to organise and execute courses of action required to attain designated types of performances”*[20]. Most adults know that walking regularly is the most important form of exercise for adults aged 65 years and older is an important aspect to living a healthy life [21]. However, simply knowing about the importance of exercise is seldom sufficient to motivate a sedentary individual to initiate and/or maintain physical activity on a regular basis [22]. Some of the major factors that increase the likelihood a person will sustain new physical activity behaviour include: social support, self-efficacy, active choices, health contracts, perceived safety, regular performance feedback and positive reinforcement [23].

The purpose of this article is to describe the protocol, theoretical rationale and intervention for this study which has been given the acronym VaLUE (Venous Leg Ulcers And Exercise). This study is a three-month clinical trial of the VaLUE intervention which will be deployed via telephone calls, to provide an engaging home based exercise program for adults with venous leg ulcers. The exercise program is unique in its focus on the importance of making regular exercise a SMART activity, which means goal setting is Simple, Measurable, Achievable, Realistic and Timed. The exercise program will teach both behavioural and problem-solving strategies for successfully establishing and maintaining realistic exercise goals for participants with venous leg ulcers.

## Methods/design

### Study design

**Figure 1:**
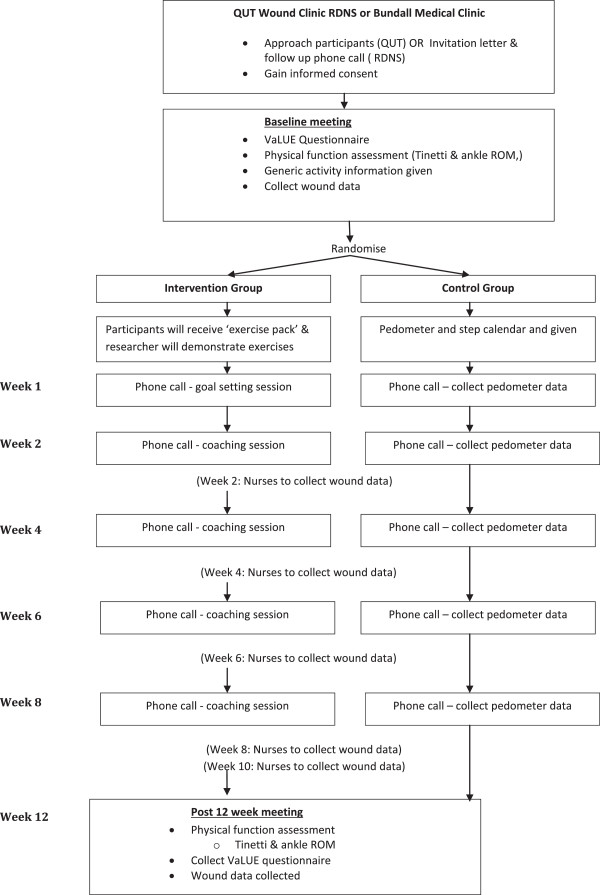
Consort flowchart of VaLUE trial.

The VaLUE study is an open, two arm, parallel group, pragmatic multi-site randomised controlled trial (RCT) to test the effectiveness of a multi-component approach to promoting exercise in adults with venous leg ulcers. The VaLUE intervention includes two components: an exercise booklet designed specifically for adults with venous leg ulcers; and a telephone-delivered behavioural exercise intervention to promote self-regulatory skills for activity behaviour change linked to the exercise booklet. Participants are randomised into the VaLUE intervention or the comparison group for 12 weeks, a CONSORT flowchart of the trial design is shown in Figure 1.

### Eligibility criteria and exclusions

To be eligible to participate in the RCT, patients have to meet the following criteria: patients with a leg ulcer of primarily venous aetiology as determined by the clinician in charge; an Ankle Brachial Pressure Index ≥0.8 and ≤1.2; able to understand English; willing to participate in a telephone intervention; and who do not have a diagnosed cognitive impairment. The exclusion criteria are: cognitive impairment; and an inability to understand English. Eligible participants are approached by a research assistant and provided with an Information Package and Consent form. Participants receive no incentives.

### Recruitment, screening and informed consent

Participants with open venous leg ulcers are recruited from three clinical sites in Australia, to facilitate generalisability of the results and to ensure timely recruitment. All study procedures have been approved by QUT’s University Human Research Ethics Committee (UHREC) and Royal District Nursing Service (RDNS) Human Research Ethics Committee (HREC) and the Private General Practice at Bundall Medical Centre. All eligible participants within each wound clinic are assessed for inclusion in the study. All participants are asked to provide written informed consent prior to enrolment in the study. For adults with more than one venous leg ulcer the larger of the ulcers is included as the study ulcer. The protocol conforms to CONSORT guidelines for reporting non-pharmacological interventions and has been registered with the Australia and New Zealand Clinical Trials Registry (ANZCTR) prior to study commencement.

### Randomisation and blinding

After completing all baseline data collection measures, participants are randomised to either intervention group protocols or control group protocols for 12 weeks or until healed. Participants in the intervention group receive evidence based wound care, and a VaLUE pack which includes a pedometer, general advice for looking after their legs and an information sheet. In addition those in the intervention group receive an instructor developed exercise booklet ‘VaLUE your Independence’, along with a verbal explanation and demonstration of the resistance exercise program. Those in the intervention are followed up by the first author for telephone exercise coaching at week one, two, four, six eight and twelve. Participants in the control group will also receive a VaLUE pack which includes a pedometer and the same general advice information sheet for looking after your legs.

The randomisation schedule has been prepared by an assistant independent to the research project using a computer generated random numbers table. To conceal randomisation, the independent staff member prepared consecutively numbered, sealed, opaque envelopes for each site. The envelopes are kept in a locked location at each site accessible only by an unblinded researcher. As VaLUE is a self-administered intervention, blinding of the participant and the researcher who conducts the telephone follow-up is by necessity unblinded.

### Intervention

#### VaLUE program

The VaLUE intervention has been informed by Social Cognitive Theory (SCT). SCT focuses on the complex, dynamic relationships between the individual, health behaviours and their environment, where all elements interact and influence each other [15]. This theory postulates that people’s beliefs about their capabilities are a better predictor of their behaviour than are their actual capabilities. For example, people with high self-efficacy regard tasks as a challenge rather than a risk, setting goals for themselves and staying committed to them. People with low self-efficacy avoid difficult tasks; they have low aspirations and a weak commitment to their goals [24]. To increase self-efficacy, strategies to improve goal achievement is embedded throughout the intervention. The VaLUE intervention is based on the SMART concept, therefore assisting individuals to improve their exercise self-efficacy. Specific behaviour change techniques included are the provision on the link between walking, leg exercise and CVI, setting graded tasks (pedometer step counts), identifying barriers and ways to overcome them, prompting self-monitoring by use of the pedometer, identifying social support and relapse prevention. Table 1 lists the theory-based behavioral strategies which are supported in the VaLUE intervention.

**Table 1:** Description of behaviour change techniques targeted in consultations

**Behaviour change technique**	**How the technique was used in the consultation**
Motivation for taking part	Discussion of why participant interested in the project
Information on the link between walking/leg exercises and health	Reflection on role of physical activity for health and well being
Self-reflection on pros and cons of increasing walking and leg exercises	Weighing up pros and cons of introducing leg exercise and starting walking
**Graded tasks**	
Instruction	Leg exercises and using the pedometer
Self-monitoring	Using the pedometer to monitor progress towards goals
Identifying and overcoming barriers	Recognising barriers and inviting participants to consider ways to overcome
Relapse prevention	Recognising situations, thinking of alternatives

At baseline, participants in the intervention group receive a VaLUE pack which includes printed information that details important information about exercise and venous leg ulceration. The exercise booklet includes general information about the benefits of exercise and common barriers to physical activity and solutions. The booklet has detailed information about stretching, walking recommendations and lower leg resistance exercises designed to improve the strength and endurance of the calf muscle.

Participants in the intervention group are provided with up to 6 individual phone calls throughout the 12-week intervention. Upon recruitment participants nominate their time preference for receiving their telephone coaching session. In accordance with recent recommendations for chronic disease management, and as used in our pilot study [11] the intervention is staged-adapted and participants are encouraged to exercise within their own capabilities, which is influenced by individual symptomatology. An exercise physiologist conducts the delivery of the intervention. Treatment commences within one week of the baseline assessment.

### Exercise components

*Lower limb resistance exercises* (5–6 sessions/week) are strongly encouraged, as strength training has benefits of improving the capacity of the calf muscle pump to return blood to the heart and lower venous hypertension [25,26] and is consistent with exercise guidelines for older adults [27]. In the exercise intervention booklet, participants are provided with detailed pictures and instructions, and guidelines on the number of sets and repetitions of each exercise, along with options for progression. The exercise protocol is detailed in Additional file 1, the program has been designed to be self-managed therefore all participants will be advised to begin at the same point, i.e. stage 1 level 1 and self-progress after satisfactorily completing that level for 3 days consecutively before advancing to the next. The lower limb exercises do not require any additional equipment.

### Walking

A target of 150 minutes (30 minutes over 5 days per week) of low- moderate intensity, (as defined by a score between 9 – 14 on the Borg scale [28]) planned activity is encouraged, consistent with management goals for older adults [29]. As most participants are relatively inactive at baseline, increases in exercise are initially small and gradually increase towards the goal of 30 minutes per day of low-moderate intensity.

### Exercise logs

Participants log their exercise (e.g., number of repetitions, sets, frequency and exertion level) in their participant manual in each week to reinforce their sense of mastery over time.

### Education/behaviour change

Behaviour change strategies and principles used to guide the intervention are guided from SCT. The health education component of VaLUE is designed to increase self-efficacy for exercise, exercise adherence and self-efficacy for management of other VLU related symptoms. The exercise coaching sessions address: the use of exercise to manage symptoms and improve healing opportunities, basic elements of a balanced exercise program, how to exercise, barriers to exercise maintenance, and strategies to overcome barriers (Table 1). The health education component occurs during the first education session and usually lasts for 20–30 minutes. Behaviour change skills emphasised in this intervention include self-monitoring, goal setting and positive reinforcement, positive self-talk, self-reward, motivational advice and/or relapse prevention coaching.

In the VaLUE study participants are provided with self-monitoring tools; a pedometer (Yamax SW500) to monitor daily steps, and ‘keep it up’ worksheets, to monitor their daily lower limb exercises and walking steps respectively. Participants receive a detailed workbook at the start of the trial and up to 6 telephone calls over 2 months to support initiation and maintenance of exercise. Call frequency is flexible, so that it can be tailored to meet the needs of the participant. For example, an extra weekly call could be made during a period of relapse.

Participants work with the first author to identify small, gradual changes to exercise patterns that are able to be mastered and maintained, rather than drastic changes which are less likely to be maintained over time. This facilitates a sense of mastery and confidence that can be built upon throughout the intervention and hence build self-efficacy. Each intervention contact results in a behaviourally specific action plan that specifies exactly what the participant intends to do and when; barriers and supports are identified; confidence assessed and problem-solving is discussed as necessary. These steps are repeated during intervention calls, with goals being adjusted as necessary. Participants are encouraged to set their own goals based on their fitness level and circumstances.

### Telephone reinforcement for VaLUE

All telephone calls are provided by the first author who has qualifications in exercise physiology and nursing and follow a set telephone protocol. The first author reviews participants’ original or amended goals, participation in exercise program since the previous call, barriers experienced, motivation for exercise, recent symptoms and wound status. Adverse events are documented. If participants are experiencing difficulties, the calls address barriers and ways to reactivate participation. Each follow up call lasts approximately 10 – 15 minutes.

### Program adherence

Participants inform the investigator of requests for days and times to receive their phone calls during the eligibility screening phase. Adherence is categorised as follows: self-reported participation in 75% or more of the sessions is ‘excellent’; participation in 50–74% of sessions is ‘good’; participation in 25–49% of sessions is moderate; and participation in less than 25% of the sessions is ‘poor’.

### Treatment fidelity

Treatment fidelity is monitored to ensure the phone calls are consistently delivered as intended. Treatment fidelity is considered in the following aspects; a clearly written protocol in relation to the telephone coaching and the call duration, is documented.

### Usual care group

In order to minimise attrition over the 3 month study duration, participants in the usual care group receive a VaLUE bag with a generic information brochure “Taking care of your legs”, a pedometer and ‘working at it’ sheets. The usual care group receive phone calls at the same time points as the intervention group, however they are not provided with any goal setting strategies. If at any point a participant in the usual care group is to ask for exercise advice they are referred to the wound care nurses in the clinic to discuss standard advice.

### Data collection and outcomes

#### Procedures and data collection

All staff of the respective trial sites have been trained on the study protocol, informed consent and assessment data collection procedures. A data collection training manual has been developed to guide research staff through step-by-step administration of informed consent and data collection procedures. Assessments are completed in person at baseline and at 12 weeks (Figure 1).

Potential participants complete a brief screening, either in person or by telephone. During the screen, an in-person baseline interview is scheduled for eligible participants. Informed consent is obtained before the interview, and all questionnaires are interviewer-administered. Most baseline and follow-up interviews are conducted at the intervention site, but some are conducted in the patients’ home.

Baseline descriptive demographic information is collected on health and medical history; venous history and previous ulcer characteristics, clinical status, functional and general health status. A VaLUE questionnaire is administered at baseline and week 12, which collects data related to the social-cognitive constructs which underpin the intervention and to assess self-efficacy, outcome expectations, and a range of dimensions related to health outcomes as shown in Table 2. A functional assessment is conducted only at baseline and week 12, including: range of ankle motion and a balance and walking measure. Wound data for each participant is collected by the respective clinical staff fortnightly at each site; measures such as ulcer area and the skin integrity are documented. Clinical staff at all sites have been trained in data collection for the study for consistency of data collection. Data related to intervention delivery is tracked by the first author following each call. This includes data on call outcomes (call completion versus missed calls), call duration, call content (via a checklist of topics) and self-reported data on exercise adherence.

**Table 2:** Study procedures for both intervention and control group

**Measure**	**Week**						
	**0**	**1**	**2**	**4**	**6**	**8**	**12**
Clinical history	×						
Sociodemographic data	×						
***Wound healing***							
Pressure Ulcer Healing Score (PUSH)	×		×	×	×	×	×
Ulcer characteristics, level of compression	×		×	×	×	×	×
***Physical activity outcomes***							
Yale Physical Activity Survey	×						×
Adherence to exercise program	×	×	×	×	×	×	×
Pedometer		×	×	×	×	×	×
***Functional outcomes***							
Lawton Instrumental ADL	×						×
Tinetti Gait and Balance Test	×						×
Ankle Range of motion	×						×
**Psychosocial beliefs toward exercise**							
Exercise Self-efficacy Scale	×						×
Outcome expectancies for exercise	×						×
Fear avoidance beliefs for exercise	×						×
***Health-related quality of life***							
The MOS Social Support Survey	×						×
The Medical Outcomes Study (MOS) Pain Measures	×						×
Medical Outcomes Survey Short Form-8 questionnaire (SF-8)	×						×
The Geriatric Depression Scale (GDS)	×						×

### Primary outcome measures

*Incidence of complete wound closure* at the completion of the study (12 weeks after initiation of exercise intervention). Wound photographs and tracings are collected fortnightly or monthly by the wound care nurses via the use of acetate grids and Visitrak for planimetry (Smith & Nephew Medical Limited, Hull, England) to determine: ulcer area and fortnightly percent reduction in area; time to healing; and incidence of complete wound healing. Healing is defined as full epithelialisation of the ulcer which has been maintained for at least two weeks without breakdown. Visitrak has been validated as a reliable measure of ulcer size with high intra-inter reliability [30]. The Pressure Ulcer Scale for Healing (PUSH) [31] tool is a standardised method of assessing and monitoring the severity of both pressure ulcers and venous leg ulcers using the following parameters: length, width, amount of exudate and wound bed tissue type to track healing progress over time. The PUSH tool has established psychometric properties, such as validity and sensitivity for venous wounds [32].

### Secondary outcomes

#### Physical activity

*Self-reported physical activity* is measured through eight questions from the Yale Physical Activity Survey (YPAS) [33]. The questions measure the participant’s physical activity over the previous week undertaking exercise, household and recreational activities. Moderate to high validity and reliability have been established in a number of studies [34,35].

*Exercise adherence data* is obtained from the researcher’s data sheets recorded on paper during phone calls and home-based participants’ self-report exercise logs, which provide important information about whether participants are exercising at home.

*Objective step data* is collected at baseline (7 days of step data – minimum of 3 days required) to determine any baseline differences and at each phone call timepoint.

### Functional ability

The Tinetti gait and balance test for agility is assessed at baseline and week 12, this tool is a reliable and valid clinical test to measure gait and balance in older people and other patient populations [36]. The Tinetti was found to have the best predictive validity for fall risk in elderly people when compared to the timed up and go, functional reach test and one-leg stance test. The test is easily administered and provides information about an individual’s ability to ambulate and transfer safely [36].

Ankle range of motion using a goniometer (Baseline plastic 360°) is used for collecting data on range of motion (ROM) of the ankle joint using a standardised protocol across sites. Participants are tested for ankle plantar flexion and dorsiflexion at baseline and 12 weeks after recruitment. Participants are asked to maximally perform ankle dorsiflexion and plantarflexion; the clinician then adds the two scores for the total range of ankle motion.

Lawton’s Instrumental Activities of Daily Living (IADL). The instrumental activities of daily living are assessed at baseline and week 12. These activities are more complex than basic activities of daily living, and being able to perform them allows a person to be independent within a community. The survey is designed to assess a patient’s everyday functional competence with consideration to housework, tasks involving mobility, managing the home and property; catching the bus; cooking meals and going shopping, use of medicine and financial behaviour [37]. Few studies have tested the psychometric properties of the Lawton IADL scale, although it has been used for almost 40 years. Lawton and Brody originally determined acceptable levels of inter-rater reliability and construct validity for the Lawton IADL scale by examining its correlation with other scales that measure functional competence.

### Health-related quality of life

SF-8™ Health Survey1Medical Outcomes Survey Short Form-8 questionnaire (SF-8) is measured at baseline and week 12, it is a widely used, valid and reliable measure to assess health related quality of life [38].

The MOS Social Support Survey is measured at baseline and week 12, it examines the perceived social support available to patients and represents the multiple dimensions of support including emotional, tangible, affectionate, and positive social interaction [39].

The Geriatric Depression Scale (GDS) is measured at baseline and week 12, it is used to assess depressive symptoms over the last four weeks [40]. The five-item GDS has been validated as an effective screening tool of depression in cognitively intact older subjects [41] and reliable in a variety of settings [42].

The Medical Outcomes Study (MOS) Pain Measures is measured at baseline and week 12, it is a valid and reliable means of assessing intensity, frequency and duration of pain on behaviour and mood [43].

### Psychosocial beliefs toward exercise

Self-efficacy for Exercise scale is measured at baseline and week 12, it asks the participants about their confidence in performing exercise in a number of different circumstances [44] that has been previously validated in older populations.

The Outcome Expectancies for Exercise Scale is measured at baseline and week 12, d and has been included in this analysis due to the importance of including outcome expectations, especially with older adults when measuring self-efficacy [45].

Fear-Avoidance Beliefs Questionnaire (FABQ) [46] is measured at baseline and week 12. The FABQ is a sixteen item self-report questionnaire aimed at quantifying the beliefs of how work and physical activity affect pain and whether they should be avoided. The two subscales, fear-avoidance beliefs for work (FABQ-work) and fear-avoidance beliefs for physical activity (FABQ-physical), this scale has been used previously in venous leg ulcer patient populations [47].

### Data analysis

Differences in primary and secondary outcomes between groups will be compared using intention to treat analysis. Once a participant is randomised to a study group, they will be considered a trial participant and analysed according to their allocated group, regardless of missing data for follow ups or the amount of intervention received. Bivariate relationships will be tested with Pearson or Spearman correlations, independent t-tests or Mann–Whitney U tests to examine relationships between the dependent variables and the independent variables or chi square for categorical varibales. Each primary and secondary outcome will be modelled using mixed linear models to determine differences between groups and adjust for potential confounders. Potential confounders such as baseline characteristics, for example disease severity, ulcer size and/or duration etc. will be included and controlled for in the final model. Data analysis will be performed using SPSS v21. Statistical significance will be set at p < 0.05 for all analyses.

### Sample size calculations and expected loss to follow up

The sample size estimation was based on wound healing data from our pilot study and an estimated post-intervention difference in healing between groups. A sample of 44 participants randomised to each group will be sufficient to detect a moderate effect size difference (80% power and a 5% significance level) of 20% difference in proportions of healed ulcers between groups after a 12 week exercise intervention. This figure rises to 55 in each group to allow for a 20% loss to follow-up.

## Discussion

Venous leg ulceration affects a significant proportion of the population over 60 years [1] and the clinical symptoms of the disease impose a significant burden to patients, the healthcare system and wider economy. The VaLUE study aims to provide evidence for the effectiveness of a home based pragmatic exercise program to improve physical activity and therefore venous return, thereby promoting healing for adults with venous leg ulcers. This study uses a telephone based intervention to educate, motivate and change behaviours based on SCT that support increases in physical activity. Findings from this study will add to the growing research literature on the health benefits of exercise for chronic disease management. Furthermore this study may yield further insights to our understanding of the strengths and limitations of behaviour change in relation to a home based exercise program, identifying those groups of patients who benefit most or least from this type of an intervention.

A self-management intervention that can improve an individual’s exercise self-efficacy and self-management capacity could have potential significance in improving the management of people with chronic venous leg ulcers in the community.

### Trial status

Current follow up.

## Competing interests

The authors declare that they have no competing interests.

## Authors’ contributions

JOB, KF, HE and GK contributed to conception and design of the study. JOB registered the trial, and is implementing the study. KF, HE and GK have been involved in monitoring at all stages. All authors have contributed to drafting the manuscript, read and approved the final version.

## Pre-publication history

The pre-publication history for this paper can be accessed here:

http://www.biomedcentral.com/1471-5945/14/16/prepub

## Supplementary Material

Additional file 1 Exercise Protocol. Click here for file
