# Supplementary material for: Testing the effectiveness of a self-efficacy based exercise intervention for adults with venous leg ulcers: protocol of a randomised controlled trial
Source: BMC Dermatol. 2014 Oct 3;14:16. doi: 10.1186/1471-5945-14-16 (PMC4188410; doi:10.1186/1471-5945-14-16)
Supplement: Additional file 1 — Exercise Protocol. [file 1471-5945-14-16-S1.pdf]

Stage 1. Seated heel-rises (both legs)

- Level 1 10 x 3 sets 3 times per day everyday
- Level 2 15 x 3 sets 3 times per day everyday
- Level 3 20 x 3 sets 3 times per day everyday
- Level 4 25 x 3 sets 3 times per day everyday

Stage 2. Standing heel-rises (both legs)

- Level 1 10 x 3 sets 3 times per day everyday
- Level 2 15 x 3 sets 3 times per day everyday
- Level 3 20 x 3 sets 3 times per day everyday
- Level 4 25 x 3 sets 3 times per day everyday

Stage 3. One legged heel-rises

- Level 1 10 x 3 sets 3 times per day everyday
- Level 2 15 x 3 sets 3 times per day everyday
- Level 3 20 x 3 sets 3 times per day everyday
- Level 4 25 x 3 sets 3 times per day everyday
